# Microalgae for High-Value Products Towards Human Health and Nutrition

**DOI:** 10.3390/md17050304

**Published:** 2019-05-24

**Authors:** Ines Barkia, Nazamid Saari, Schonna R. Manning

**Affiliations:** 1 Department of Food Science, Universiti Putra Malaysia, Selangor 43400, Malaysia; ines.barkia@gmail.com; 2 Department of Molecular Biosciences, UTEX Culture Collection of Algae, University of Texas at Austin, Austin, TX 78712, USA; schonna.manning@utexas.edu

**Keywords:** Microalgae, bioactive compounds, health benefits, antioxidant activity, antihypertensive activity, anti-inflammatory activity

## Abstract

Microalgae represent a potential source of renewable nutrition and there is growing interest in algae-based dietary supplements in the form of whole biomass, e.g., *Chlorella* and *Arthrospira*, or purified extracts containing omega-3 fatty acids and carotenoids. The commercial production of bioactive compounds from microalgae is currently challenged by the biorefinery process. This review focuses on the biochemical composition of microalgae, the complexities of mass cultivation, as well as potential therapeutic applications. The advantages of open and closed growth systems are discussed, including common problems encountered with large-scale growth systems. Several methods are used for the purification and isolation of bioactive compounds, and many products from microalgae have shown potential as antioxidants and treatments for hypertension, among other health conditions. However, there are many unknown algal metabolites and potential impurities that could cause harm, so more research is needed to characterize strains of interest, improve overall operation, and generate safe, functional products.

## 1. Introduction

Algae are a complex, polyphyletic assemblage of (mostly) photosynthetic organisms. Organisms in the algae include diverse micro- and macroscopic forms that are distributed across the tree of life, including eukaryotic and prokaryotic members [1], making algae the most genetically-diverse set of organisms on the planet. While the phylogenetic relationships continue to be resolved among groups, algae can be broadly assigned to eleven major phyla: Cyanophyta, Chlorophyta, Rhodophyta, Glaucophyta, Euglenophyta, Chlorarachniophyta, Charophyta, Cryptophyta, Haptophyta, Heterokontophyta, and Dinophyta.

The major groups of eukaryotic algae arose through multiple endosymbiotic events, resulting in broadly-distributed and highly-diverse lineages. The Glaucophyta, Rhodophyta, and Chlorophyta evolved via the primary endosymbiosis of a photosynthetic cyanobacterium (Cyanophyta), which gave rise to the chloroplast. Secondary endosymbiosis of green algae led to two major groups, the Euglenophyta and photosynthetic Rhizaria, the Chlorarachniophyta [2]. Similarly, the secondary endosymbiosis of a red alga gave rise to the Cryptophyta, Heterokontophyta, Haptophyta, and Dinophyta. There are tertiary (and possibly quaternary) endosymbiotic events between the Dinophyta and Haptophyta, resulting in genomes greater than 500 Gbps [3,4].

The pigments found in the algae are diagnostic for each major group [5]. Cyanobacteria contain chlorophyll-a, -d, and -f, as well as the phycobiliproteins, phycocyanin, allophycocyanin, and phycoerythrin. Glaucophytes contain chlorophyll-a and harvest light via phycobiliproteins. The Chlorophyta contain chlorophyll-a and -b, as well as a suite of carotenoids, including β-carotene and various xanthophylls (e.g., astaxanthin, canthaxanthin, lutein, zeaxanthin, etc.). The primary pigments of the Rhodophyte algae are phycoerythrin and phycocyanin, which can mask chlorophyll-a. There are no accessory chlorophylls; although, red algae do produce a broad spectrum of carotenes and xanthophyll light-harvesting pigments.

**Figure 1:**
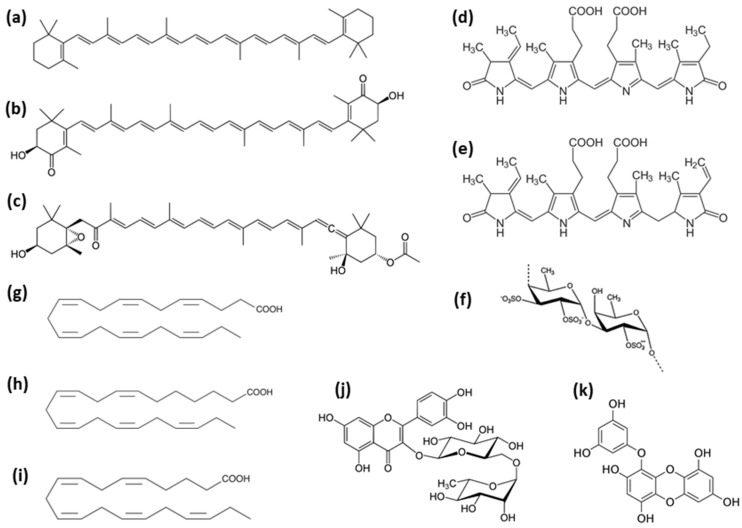
Bioactive compounds from microalgae include: carotenoids, β-carotene (**a**). astaxanthin (**b**), and fucoxanthin (**c**); phycobilins, phycocyanin (**d**) and phycoerythrin (**e**); sulfated polysaccharides (**f**); omega-3 fatty acids, docosahexaenoic acid (**g**), docosapentaenoic acid (**h**), and eicosapentaenoic acid (**i**); and phenolics, rutin (**j**) and eckol (**k**).

Cyanobacteria and eukaryotic microalgae also synthesize numerous secondary metabolites, including potentially beneficial bioactives, such as antioxidants, as well as many compounds that are broadly characterized as toxins. These bioactive compounds are produced via the shikimic acid, mevalonic/non-mevalonic acid, and acetate pathways, e.g., anatoxins, brevetoxins, cylindrospermopsin, microcystins, and prymnesins, among many more (see [6]). There are greater than 200 bioactive metabolites predicted from cyanobacteria [7], and thousands more are predicted from eukaryotic microalgae. However, there are very few assays or reference standards available for the detection of microalgal metabolites, indicating a major gap and a need for more research in this area. Examples of microalgal pigments and bioactive compounds discussed in this review are illustrated in Figure 1.

Microalgae are presented as new model organisms for a wide range of biotechnological applications, including biodiesel production [8], wastewater bioremediation [9], and dietary supplements for animal and human nutrition [10,11,12]. Recent economic viability studies have shown that, due to limited biomass productivity and the costs of commercial algal production, the production of biofuels is not cost-effective unless the process is coupled with the commercialization of higher-value co-products [13]. By comparison, the use of microalgae biomass for the development of products with health benefits, such as nutraceuticals and functional foods, is a rapidly growing market [14]. For example, the worldwide carotenoid market is expected to increase from $1.24 billion USD in 2016 to more than $1.53 billion USD by 2021 [15].

A lot of funding has been invested over the last few decades to screen microalgal bioactive metabolites [16]. Several bioactive compounds have been discovered and purified from marine microalgae, including sulfated polysaccharides [17], marennine [18], various carotenoids (e.g., astaxanthin, fucoxanthin, β-carotene [19,20]), omega-3 fatty acids [21], and polyphenols [22]. Some of these metabolites have demonstrated biological activities, including potent antioxidant, anti-inflammatory, anticancer, and antiviral properties [23,24,25]. Thus, microalgae and microalgae-derived compounds have great potential as supplements in the human diet for the prevention, management, and treatment of physiological aberrations, in addition to providing sustainable natural resources in lieu of synthetic dietary supplements [26]. However, there are emergent biological and economic challenges associated with the large-scale cultivation of microalgae and the biorefinery process that must be addressed to ensure the sustainable production of high-value products with nutritional and health benefits.

## 2. Microalgae-Based Functional Foods

The use of “wild” harvested macroalgae for food purposes has been well established for hundreds of years in Asiatic countries. Nevertheless, the commercial cultivation of microalgae for biomass started only 60 years ago, e.g., *Chlorella vulgaris* was produced in Japan and Taiwan in the 1960s, and the mass production of *Arthrospira* (formerly known as *Spirulina*), *Dunaliella salina*, and the cultivation of *Haematococcus pluvialis* was developed in the USA, Israel, Australia, China, and Thailand in the 1980s [27]. The market for dietary supplements in the form of dried biomass is presently dominated by *Arthrospira* and *Chlorella* (Table 1).

Interest in whole-biomass products, commercially known as “super foods”, are attributed to reports of high protein content, nutritive value, and health benefits [29]. *Arthrospira* is marketed as a source of high-quality protein, γ-linolenic acid, and phycocyanin content. *Arthrospira* is also reported to exhibit antiviral, anticancer, antioxidant, and anti-inflammatory properties among other effects [30,31,32]. Green algae in the genus *Chlorella* have been advertised as providing a “growth factor”, which is a water-soluble extract composed of a variety of substances, including nucleic acids, amino acids, vitamins, minerals, polysaccharides, glycoproteins, and β-glucans [33]. Extracts from *Chlorella* have demonstrated many beneficial properties, such as lowering cholesterol as well as antioxidant, antibacterial, and antitumor activities [34,35,36].

Despite the reported benefits, there are also numerous studies indicating that the ingestion of high amounts of *Chlorella* or *Arthrospira* may result in severe side effects. For example, the excessive intake of *Chlorella* was documented as causing allergies, nausea, vomiting, and other gastrointestinal problems [37]. Some of these effects were reported with the 2016 recall of Soylent products that contained *Chlorella* algal flour as an ingredient. In other cases, *Chlorella* was found to induce acute tubulointerstitial nephritis, which can cause renal failure [38]. With excessive ingestion of *Arthrospira*, possible side effects included diarrhea, nausea, and vomiting [39]. The reported bioavailability of vitamin B_12_ has also been riddled with controversy, which makes it an unreliable supplement for treating B_12_ deficiency [40]. Moreover, Gilroy et al. reported that under certain uncontrolled cultivation conditions, *Arthrospira* biomass may contain significant amounts of heavy metals (e.g., mercury, cadmium, arsenic, and lead) or toxins, like microcystins, from co-occurring cyanobacteria, wherein prolonged consumption of *Arthrospira* tablets could cause irreversible damage to visceral organs, such as the kidneys and the liver [41].

The potentially serious side effects of commercially-accepted microalgae, like *Chlorella* and *Arthrospira*, have raised serious doubts about the suitability of other strains of microalgae for human consumption [42]. Moreover, there are many more bioactive compounds yet to be discovered. Thus, there is an immediate need to detect potentially harmful metabolites, as well as to develop methods to extract targeted compounds from microalgae instead of using the whole biomass.

## 3. Biochemical Composition of Microalgae

Microalgae produce a suite of biochemical molecules, including carbohydrates, proteins, lipids, and nucleic acids, as well as essential vitamins and minerals. The cellular content of each fraction varies according to the specific strain of algae and their physiological responses to biotic and abiotic factors, e.g., light intensity, photoperiod, temperature, nutrients, and growth phase [43,44]. The primary biochemical compositions of selected species of microalgae are shown in Table 2.

### 3.1. Proteins

Proteins play an important role in the structure and metabolism of microalgal cells. They are an integral component of the membrane and light-harvesting complex, including numerous catalytic enzymes involved in photosynthesis [57]. The protein content of many microalgal species can compete, quantitatively and qualitatively, with conventional protein sources [58,59]. In terms of quantity, several species of microalgae are reported to possess very high concentrations of protein; this can range from 42% to over 70% in certain cyanobacteria [60,61] and up to 58% for *Chlorella vulgaris* on a dry weight basis [47]. In terms of quality, microalgae contain all of the essential amino acids that mammals are unable to synthesize. Moreover, the amino acid profiles are well-balanced and similar to high-quality protein sources, such as lactoglobulin, egg albumin, and soy [57]. The application of microalgal proteins has been limited in foods thus far. This is mainly due to the presence of non-protein components (e.g., chlorophyll) that can affect the color and taste of the microalgae-based products [47]. Moreover, the rigid cell wall of some strains, mainly green microalgae (e.g., *Chlorella* and *Tetraselmis*), can lower the extraction efficiency of intracellular proteins and whole biomass applications can result in low digestibility in humans (see later) [47,62].

### 3.2. Carbohydrates

Carbohydrates, including mono-, oligo-, and polysaccharides, have both structural and metabolic functions. Carbohydrates can be found attached to proteins or lipids (e.g., glycoproteins and glycolipids), and complex polysaccharides compose the major structural features of the cell wall [63]. In addition, microalgae make glucose and starch-like energy storage products, which are the primary carbon-containing products from photosynthesis [57].

Polysaccharides are produced in a variety of forms according to the group of microalgae. Cyanophytes are known to accumulate glycogen, although some species synthesize semi-amylopectin [64]. The Chlorophyta synthesize starch in the form of two glucose polymers, amylopectin and amylose [65], while Rhodophyta produce a carbohydrate polymer known as floridean starch [66]. Diatoms (Bacillariophycae, Heterokontophyta) produce chrysolaminarin, a linear polymer of β(1,3) and β(1,6) linked glucose units [67]. Some diatoms can store up to 30% of their dry weight as (1,3)-β-D-glucan during the exponential growth phase and cells can accumulate up to 80% under strong nutrient limitations [68]. Similarly, a strain of *Tetraselmis suecica* was reported to accumulate between 11% and 47% of its dry weight as starch in nutrient replete vs deplete conditions, respectively [69].

While microalgae represent a source of beneficial carbohydrates, their use in food applications has been very limited. Instead, microalgal polysaccharides are gaining more importance in the cosmetic industry as hygroscopic agents and antioxidants for topical applications, including lotions and creams [17].

### 3.3. Lipids

Among the chemical components, lipids have received the greatest attention for extraction and commercialization. When research on algal lipids first began, the primary goal was the production of biodiesel. However, polyunsaturated fatty acids, i.e., omega fatty acids, have considerably more commercial value as nutraceuticals and in infant formulations [70].

Lipids provide the structural components of plasma membranes and neutral lipid bodies function as energy reservoirs secondary to polysaccharide stores. The lipid fraction of microalgae is mainly composed of (i) neutral lipids that include acylglycerols, free fatty acids, and carotenoids (e.g., β-carotene), and (ii) polar lipids, such as various phospholipids and galactolipids. Generally speaking, most microalgae are rich in polar lipids in the exponential phase of growth, and they accumulate triacylglycerols under stress conditions, which is typically during the stationary phase when nutrients are limited [71,72]. The fatty acid profile of microalgae is generally characterized by a mixture of C16 and C18 saturated and unsaturated fatty acids as well as longer carbon-chain lengths, including many omega fatty acids. Saturated fats are usually stored in neutral lipid bodies, whereas unsaturated fatty acids are mostly associated with polar lipids in the various membranes that serve to maintain membrane fluidity under varying cultivation conditions [57].

The lipid content of many microalgal species is well documented, and this fraction can represent anywhere from 20% to 50% of the dry biomass (w/w). However, other values ranging from 1% to 70% have also been reported [12]. The production of lipids depends on the microalgal species, and the synthesis of different types of lipids is greatly affected by cultivation conditions, i.e., growth phase, nutrient availability, salinity, light intensity, temperature, and pH [73,74]. It is well known that the intracellular lipid content increases significantly during nitrogen limitation, and cultures in the stationary phase have been shown to double their neutral lipid content and synthesize polysaccharides at the expense of proteins [44,75].

### 3.4. High-Value Natural Products

Many natural products from microalgae have attracted special attention due to their broad spectrum of biological activities. According to the review of [76], more than 4000 studies on bioactive compounds from microalgae were published in the years between 1926 and 2016. From an industrial perspective, these types of products have much higher economic value than whole, dried microalgal biomass even if their production volumes are smaller (Table 3).

The most popular microalgal products on the market are pigments and fatty acids [80]. More than 1200 tons of β-carotene were produced in 2010 [10]; this pigment is extracted from *Dunaliella salina*, a green alga that grows in open ponds under high salinity and light conditions [81]. Another commercially-important carotenoid, astaxanthin, is synthesized by the freshwater green alga, *Haematococcus pluvialis* [82]. While there is currently less production of astaxanthin than β-carotene, it has a higher market value [77]. However, the commercialization of astaxanthin has been slower because of the higher production costs and the absence of a pre-established market as a nutraceutical for human use [14].

Polyunsaturated fatty acids (PUFAs) from microalgae are also considered among the major commercial high-value products with an estimated market value of $140 USD/kg [14]. The interest in fatty acids from microalgae is mainly attributed to their lower levels of contamination (e.g., methyl mercury, dioxins, and polychlorinated biphenyls) when compared to fish oils [83,84]. Moreover, the use of algal fatty acids has also shown beneficial effects against inflammation and a wide range of cardiovascular diseases (e.g., hypertension, cardiac arrhythmia, myocardial infarction, and thrombosis) [85,86]. Several species of microalgae (e.g., *Cryptothecodininum cohnii* and *Schizochytrium limacinum*) have been cultivated industrially in fermenters (i.e., heterotrophically) for the production of docosahexaenoic acid (DHA) [70,87]. This omega-3 fatty acid is widely used as a nutritional supplement in infant formula with an estimated worldwide wholesale market of around $9.0 billion USD/year [88]. Besides carotenoids and PUFAs, numerous high-value compounds remain to be characterized in microalgae, expanding opportunities for the discovery of new bioactive products (Table 4).

## 4. Microalgal Mass Cultivation

The mass cultivation of microalgae has been evaluated for the production of sustainable biomass for food, feed, chemicals, biofuel, and high-value products [112]. Currently, there are two primary types of mass-cultivation systems: (i) Photobioreactors (PBRs), and (ii) raceway ponds. Closed PBRs offer better control over contamination and physiochemical conditions [55]. Although, the capital, operational, and energetic costs remain significantly higher than open raceway ponds, which are much less complex and require a lower capital investment [113]

While open ponds are deemed the only economically-viable cultivation system for producing lower-cost microalgal biomass, they are prone to emergent challenges, including lower productivity and biomass yields coupled with contamination [114]. As a rule, microalgae cultivated in open raceway ponds have much lower biomass productivities when compared to laboratory cultures and closed PBR systems. For example, open raceways ponds in southern Spain had biomass productivities of 27 tons ha^−1^ year^−1^ while closed systems produced between 34–61 tons ha^−1^ year^−1^ [115].

Some of the common stress factors that affect cellular growth and physiological performance include: contaminants and grazers; availability of nutrients and CO_2_ [116]; self-shading from high cell density [117]; excess accumulation of O_2_ during the day causing photooxidation and death [118]; and significant shifts in culture pH as the microalgae absorb all of the dissolved CO_2_ during the day and release CO_2_ at night [119]. To overcome the multitude of factors associated with trying to maintain mono-algal growth, extreme cultivation conditions (e.g., high salinity and alkalinity) can be applied to exclude competing organisms; however, only a limited number of microalgae are able to be cultivated under such selective conditions [120].

Paradoxically, in nature, large blooms of microalgae occur in complex environments with varying temperatures and nutrient conditions in highly-diverse biological communities. During a bloom, microalgal species temporarily outcompete others by rapidly dividing to reach dense cell concentrations, nearing the theoretical maximum level of growth rates (μ > 1) [121]. While it may be advantageous to mimic natural conditions and grow several strains of microalgae together (polyculture), commercial growth schemes have generally focused on the cultivation of one strain, unialgal monoculture [122].

In an attempt to replicate natural bloom conditions, an efficient solution was suggested by Jovine [123] that consisted of cultivating microalgae in connected raceway ponds of increasing dimensions using reduced amounts of nutrients and without CO_2_ addition. The transfer from one pond to another was accompanied by diluting cultures with fresh medium at a dilution rate of at least two. Dilution improved the light availability in the raceway ponds and thus enhanced photosynthetic efficiency [70]. However, the dilution rate should always remain below the maximum specific growth rate of microalgae ($\mu_{max}$) in order to prevent “washout” of the cells [70]. Light and temperature also play significant roles in the growth rates of outdoor pond cultures [124,125].

It was proposed that feeding and harvesting of microalgae should be performed only during the day to prevent the loss of biomass at night due to oxidative respiration. Using a model developed by Huesemann et al. [126], researchers can now use this tool to predict biomass productivity in outdoor ponds under nutrient-replete conditions, diurnally-fluctuating light intensities, and temperature. The model has been validated for three different species of microalgae (*Chlorella sorokiniana, Nannochloropsis salina, Picochlorum* sp.), but hypothetically, it can be used to predict the biomass concentration of any new strain. The inputs required are strain-specific parameters (i.e., growth rate, μ), biomass loss rate in the dark ($\mu_{dark}$), scatter-corrected biomass light absorption coefficient ($k_{sca}$), as well as the sunlight and water temperature data. The application of this model can greatly reduce the requirement for outdoor pond tests, which represents a major bottleneck in the industrial-scale production of microalgal biomass.

## 5. Purification of Bioactive Compounds from Microalgae

### 5.1. Lipids

Various extraction and purification methods are used to recover targeted bioactive compounds from microalgal biomass. The extraction of carotenoids, including carotenes and xanthophylls, typically involves biomass pretreatment steps (e.g., acid-base treatment, enzyme lysis, mechanical disruption) to breakdown the cell wall and membranes [127]. Subsequently, carotenoids are separated from the solid biomass using traditional solvent-based extraction techniques [128,129] or more environmentally-friendly alternatives, such as supercritical fluid extraction, microwave-assisted extraction, ultrasound-assisted extraction, or enzyme-assisted extraction (for more details, see the review from Ventura et al. [130]). Finally, depending on the intended applications, additional purification steps (e.g., preparative chromatography) may be needed to concentrate and purify targeted fractions and remove contaminating residues.

Similar to carotenoids, the extraction of PUFAs requires a cell disruption treatment to maximize oil recovery [128,131]. The extraction of lipids from microalgal cells is typically performed using non-polar organic solvents or solvent mixtures, such as chloroform–methanol and hexane–isopropanol [42,132,133]. Following extraction, additional processing steps, including fractional distillation or winterization, are used to separate PUFAs from the total lipids. However, this fraction is still not suitable for human consumption due to the presence of impurities, odor, taste, and cloudy appearance. Thus, further purification, i.e., filtration, bleaching, deodorization, polishing, and antioxidant addition, is needed to enhance the quality and shelf-life of PUFAs [134,135]. It is important to note that the use of large amounts of solvents for the extraction process has raised serious concerns about health, safety, and the environment [136]. Therefore, more efforts are needed in the areas of solvent recycling, replacing traditional organic solvents with green solvents, and the implementation of more sustainable processes.

### 5.2. Polysaccharides

The extraction of polysaccharides is commonly performed using hot water [137]. This method has the advantage of simplicity and ease of scalability, but it is also time-consuming and has a low extraction efficiency [138]. Therefore, several novel extraction techniques have been applied to increase the yield of polysaccharides from cell culture. Among these novel techniques, microwave-, ultrasonic-, and enzyme-assisted extractions have recently gained increasing popularity. Microwave-assisted extraction was reported to have a shorter extraction time and higher extraction efficiency when compared to the conventional method [139]. However, this process can induce considerable changes in the chemical structure of polysaccharides and it has been shown to decrease the viscosity of starch solutions [140]. Ultrasonic-assisted extraction is another advanced method with higher extraction efficiency than the conventional hot water extraction [141]. Unlike microwave-assisted extraction, ultrasound-assisted extraction did not significantly change the structure or molecular weight of polysaccharides, such as alginates and carrageenans [142].

Following the extraction of polysaccharides, some purification steps are usually required to remove interfering substances, such as low molecular weight compounds, lipids, and colored algal compounds. The purification procedures may also include the use of solvents, e.g., a mixture of methanol/chloroform/water (4:2:1; v/v/v) [143], or the use of physical approaches, such as membrane separation, ion-exchange, size-exclusion, and affinity chromatographic methods (for a review, see Xu et al. [138]).

### 5.3. Peptides

Numerous methods have been utilized to generate bioactive peptides from food proteins (e.g., chemical hydrolysis, microbial fermentation, enzymatic hydrolysis). While fermentation systems are relatively low cost and easily scalable, this process has the disadvantage of low product yields because the microorganisms used for hydrolysis can also consume the released peptides or amino acids as substrate for their own growth [144]. On the other hand, chemical hydrolysis is difficult to control since its uses strong chemical reagents with no specificity for peptide bonds. Moreover, this process may cause irreversible damage to some amino acids [145]. Alternatively, enzymatic hydrolysis has been presented as the best option to produce bioactive peptides for human nutrition [146]. This process is much easier to control, it uses mild conditions that do not alter the structure of amino acids, and it preserves the functionality and nutritive value of the end products [147]. Furthermore, it does not require the use of large quantities of organic solvents or toxic chemicals [148].

The success of enzymatic hydrolysis relies on several parameters, such as substrate specificity and hydrolysis conditions (e.g., temperature, pH, enzyme/substrate ratio, reaction time, etc.; see [138] for a review). Different strategies have been employed to enhance the yield of bioactive peptides from protein sources. One approach consists of the optimization of the proteolytic enzymes or the form of their use (single or in combination with other proteases) [149]. In this regard, several enzymes with well-known specificities (e.g., papain, alcalase, trypsin, α-chymotrypsin, neutrase, pepsin) have been used to prepare peptides from microalgal biomass [150]. Other methods can be used to enhance the hydrolysis process, including the optimization of the substrate, its concentration, and the extent of its digestion. For example, thermal denaturation of the seaweed, *Palmaria palmate*, which is characterized by high levels of cell wall anionic polysaccharides, was reported to improve the yield of peptides by 64% to 96% [151].

The purification of bioactive peptides is necessary to enhance their concentration and bioactivity. Pre-treatment procedures are often applied to increase the extraction efficiency of bioactive peptides. These steps consist of concentrating proteins in mixtures by removing fats, carbohydrates, and other components from the biomass using ammonium sulphate (i.e., salting out) or solvent extraction [152]. Bioactive peptides can then be separated by a number of techniques according to their physicochemical parameters, e.g., size, hydrophobicity, and charge. Methods used for peptide isolation involve size-exclusion chromatography, reversed-phase high-performance liquid chromatography (RP-HPLC), and ion-exchange chromatography.

RP-HPLC is seen as the most powerful method for peptide purification. This technique uses a stationary phase and an aqueous mobile phase containing an organic solvent to rapidly separate peptides from a complex mixture [153]. It is also easily scalable, especially since the development of dynamic axial compression (DAC) technology for preparative columns and the availability of excellent and relatively affordable reversed-phase adsorbent materials [154]. Membrane-based separation processes, such as ultrafiltration, are based on the fractionation of peptides according to their molecular weight. Molecular weight cutoff (MWCO) membranes are commonly employed and come in a wide range of sizes (e.g., 10, 5, 3, and 1 kDa MWCO) [104].

Other purification technologies exploit the chemical properties of the targeted peptides. Electromembrane filtration is more selective than membrane filtration because it enables the isolation of bioactive peptides according to both the charge and molecular weight, and it does not require the application of pressure [155]. More recently, affinity chromatography has been demonstrated to be very effective for the purification of bioactive peptides. The principle of this method consists of the formation of specific reversible complexes between the peptides being purified and an immobilized ligand. For example, immobilized copper or nickel on solid supports have been successfully used for the purification of angiotensin I-converting enzyme (ACE) inhibitory peptides [156].

Following the purification steps, peptides can be lyophilized for storage and applications. At the industrial scale of peptide manufacturing for pharmaceutical purposes, commercial-scale tray lyophilizers are used to increase batch volumes and provide better control over the freeze-drying parameters [157]. Despite recent advances in peptide purification, commercial-scale production is still challenged by costly manufacturing processes, which may introduce modifications in the structure of peptides, resulting in alterations or loss of bioactivity [158].

## 6. Microalgal Compounds with Antioxidant Properties

### 6.1. Oxidative Stress and the Cell

Because of increasing concerns over oxidative stress-mediated diseases, many therapeutic approaches and herbal medicines have been developed to identify foods that are rich in natural antioxidants [159]. A wide variety of antioxidant compounds are employed from plants. However, marine resources are gaining more attention since the discovery of new compounds with higher bioactivities than terrestrial plants [23]. In this regard, marine microalgae have been proposed as interesting and important producers of antioxidants [24,160]. This is generally attributed to their ability to survive under extremely oxidizing conditions, which increases cellular antioxidant contents or triggers the production of antioxidant secondary metabolites [161].

Under normal physiological conditions, there is a balance (homeostasis) between the prooxidant and antioxidant chemical species; although, an imbalance in favor of prooxidants can lead to oxidative stress [162]. Prooxidants can be classified into oxygen-containing free radicals, generally referred to as reactive oxygen species (ROS), and non-free radical species [163]. The most important ROS species include superoxide (•O_2_^−^), hydroxyl (•OH), peroxyl (ROO•), alkoxyl (RO•), and nitric oxide (NO•), while the non-free radical species include singlet oxygen (^1^O_2_), hydrogen peroxide (H_2_O_2_), and hypochlorous acid (HOCl) [164].

ROS are generated during normal metabolism, i.e., through the respiratory chain in the mitochondria or during cell-mediated immune functions [165]. They can also be introduced by pollution, unhealthy eating habits, smoking, lack of sleep, and irradiation by exposure to ultraviolet (UV) light, X-rays, or gamma rays. If ROS are not effectively neutralized by cellular constituents, they can stimulate free-radical chain reactions that are responsible for cell, tissue, and gene damage [166]. Effects of oxidative stress include lipid membrane peroxidation, mitochondrial swelling and lysis, mutagenic actions, and posttranslational protein modifications [167,168]. These sorts of oxidative damage have been found to be linked with several degenerative diseases, e.g., Alzheimer’s, Parkinson’s, atherosclerosis, rheumatoid arthritis, cancer, as well as accelerated aging [169].

### 6.2. Carotenoids

Carotenoids are an integral part of the photosynthetic apparatus that include lipid-soluble carotene and xanthophyll pigments. These compounds harvest light energy, but they also behave as photoprotective agents against free radicals and harsh environmental conditions, e.g., intense solar irradiation and UV [170,171]. Carotenoids are naturally produced in a broad assortment of related pigments with varying ratios to harvest the broadest spectrum of light energy. Of note, β-carotene and astaxanthin represent over 90% of the total carotenoids in *D. salina* and *H. pluvialis*, respectively, when grown at extreme conditions.

The antioxidant activity of carotenoids is attributed to the numerous conjugated double bonds in their structure or the presence of specific groups, such as epoxy, acetyl, allene (C=C=C), or acetylene (C≡C) groups [172,173]. These chemical groups act by scavenging free radicals and singlet oxygen from reactive species, thus neutralizing them into less harmful molecules [174]. In other cases, the antioxidant activity has been reported to be independent of structure. In such cases, carotenoids can activate the transcription factor, Nrf2 (nuclear factor erythroid-2-related factor 2), which triggers antioxidant gene expression in particular cells and tissues [19].

Of note, the antioxidant activity of microalgal carotenoids was reported to be significantly higher than that of plants or synthetic analogues. The in vitro antioxidant activity of fucoxanthin, a brown xanthophyll found in golden algae, was reported to be >13 times higher than α-tocopherol, vitamin E [175]. Similarly, astaxanthin, a red keto-carotenoid, was 65 times higher than vitamin C and 100 times more effective than α-tocopherol [97].

### 6.3. Phenolics

Phenolic compounds are secondary metabolites commonly found in all terrestrial plants as well as microalgae and cyanobacteria [176]. Phenolics are typically synthesized to protect cells against pathogens and ultraviolet irradiation, and they possess a very wide range of biological activities, including antioxidant properties [22,163,177,178,179,180].

It has been demonstrated that the antioxidant activity of phenolic compounds is highly dependent on their chemical structure, characterized by an aromatic ring bearing one or more hydroxyl substituents [181]. The hydroxyl groups contribute to the antioxidant activity through their metal-chelating capability as well as electron/hydrogen donation capacity, generating radical intermediates of greater chemical stability than the initial radicals [182,183]. Moreover, the combined action of hydrophobic benzenoid rings and hydroxyl groups makes phenolics capable of inhibiting different enzymes involved in ROS generation, such as lipoxygenases, cyclooxygenase, and xanthine oxidase [163,184].

The contribution of phenolic compounds from microalgae to antioxidant activity is not very well established, like that of carotenoids. While some of the available research reported a positive correlation between the total antioxidant capacity and the whole phenolic fraction [22,185,186], other examinations presented contradictory results [182,183,187,188]. This has been attributed to several factors, such as the quality and the quantity of the phenolic compounds as well as the type of solvent used for the extraction process. Different strains of microalgae were reported to contain between 0.54 and 5.8 mg gallic acid g^−1^ dry weight (DW) when using various polar and non-polar organic solvents [180,183,186]. However, under similar experimental conditions, exceptionally higher values of phenolics were reported from *Chlorella pyrenoidosa* (13.2–25.8 mg g^−1^ DW) and from *Arthrospira platensis* (17–43.2 mg g^−1^ DW) [185]. Higher levels were also detected in extracts from *C. vulgaris* when pressurized liquid extraction (PLE) was used at elevated temperatures [189].

Generally, the use of polar solvents or a mixture of water and organic solvents allows for better extraction of broad groups of phenolics. While Hajimahmoodi et al. [186] and Machu et al. [185] found that hot water was the best solvent for the extraction of phenolic compounds, Li et al. [183] found that hexane (a very non-polar solvent) had the highest extraction yield of phenolics. However, it is likely that intermediate solvent mixtures would yield many more phenolic compounds. Furthermore, little is known about the nature of polyphenolic compounds that are present in microalgae, since only a few articles have assessed the complete profile of phenolic compounds using chromatography methods [185,190,191]. Thus, further identification of phenolic compounds from microalgae is needed to better understand their roles as potential antioxidants.

### 6.4. Peptides

Research in the area of bioactive peptides has increased significantly in the last decade. Natural peptides have reduced side effects when compared to synthetic supplements that may interfere with immune system activity and defense mechanisms, resulting in prooxidant effects [192]. The global interest in bioactive peptides has been accelerated by their wide range of applications as potential treatments for a diverse array of pathological conditions, including hypertension, inflammation, diabetes, and oxidative stress [193,194,195]. Current sources of bioactive peptides are from plants and animal proteins [196,197], but very rarely microalgae. According to the available literature, only a few genera (e.g., *Chlorella*, *Navicula*, *Tetraselmis*, and *Nitzschia*) have been investigated and these genera produce numerous bioactive peptides with antioxidant properties [193,198].

The antioxidant properties of bioactive peptides from microalgae have been examined in vitro, but also using cell-based assays. In vitro methods consist of assessing the capacity of bioactive peptides to scavenge reactive oxygen species and free radicals (e.g., 2,2-diphenyl-1-picrylhydrazyl DPPH, hydroxyl, and superoxide anion radicals), to reduce ferric ions or inhibit lipid peroxidation [199]. It should be noted that the measurement of antioxidant activity in vitro has very little similarity to the physiological antioxidant capacity, which has made it challenging to conduct in vivo studies. As a result, it was proposed that the assessment of lipid and protein peroxidation as well as DNA damage are the most appropriate biomarkers to monitor changes in antioxidant activity in vivo [200].

Enzymatic hydrolysis is the most common process for the preparation of antioxidant peptides, which have been tested in the form of crude hydrolysates or purified peptides. It was reported that crude hydrolysates from the chlorophytes, *Chlorella pyrenoidosa* [201], *Chlorella ellipsiodea*, and *Tetraselmis suecica* [202], showed strong antioxidant activity in vitro and on skin fibroblasts. The purified peptides, Val-Glu-Cys-Tyr-Gly-Pro-Asn-Arg-Pro-Glu-Phe and Leu-Asn-Gly-Asp-Val-Trp, from the pepsin hydrolysate of *Chlorella vulgaris* and *Chlorella ellipsiodea*, respectively, had strong superoxide and DPPH radical scavenging activities [203,204]. Similarly, two bioactive peptides, Pro-Gly-Trp-Asn-Gln-Trp-Phe-Leu and Val-Glu-Val-Leu-Pro-Pro-Ala-Glu-Leu, isolated from papain hydrolysate of *Navicula incerta*, demonstrated potent antioxidant properties in HepG2/CYP2E1 cells [205].

The exact mechanisms of the antioxidant action of protein hydrolysates have not yet been clearly elucidated. However, it was recognized that the antioxidant activity of peptides was positively influenced by various characteristics of their chemical structure, namely small molecular weight, the composition and sequence of amino acids, the degree of hydrophobicity, the presence of an indole/imidazole/pyrrolidine ring, along with the steric structure at the C- and N-terminal region [206,207,208]. The use of quantitative structure–activity relationship (QSAR) modeling of antioxidative peptides for scavenging radicals has provided additional evidence on the importance of the C-terminal region over the N-terminal for antioxidant potency [209].

## 7. Microalgal Compounds with Antihypertensive Properties

### 7.1. Hypertension and Heart Health

Hypertension is a major health concern worldwide. It is recognized to be the main cause of death and a leading risk factor of several chronic ailments, including cardiovascular disease, stroke, renal disease, and diabetes [210]. The regulation of blood pressure in the body involves a multitude of complex systems that are governed by ACE in the renin-angiotensin-aldosterone system. ACE catalyzes the conversion of inactive Angiotensin-I (Ag I) to Angiotensin-II (Ag II), a potent vasoconstrictor peptide that binds directly to Angiotensin II Type 1 Receptor in the vascular smooth muscle cells to induce a potent hypertensive effect [211]. Furthermore, Ag II stimulates the secretion of aldosterone in the adrenal cortex, which promotes sodium and thus, water reabsorption in the distal tubules [212]. As a result, the volume of fluid in the body increases, implying an increase in blood pressure. On the other hand, Ag II stimulates the production of the superoxide anion and hydrogen peroxide in the polymorphonuclear leucocytes, which elicits an endothelium-dependent vasocontraction effect through the inactivation of endothelium-derived relaxing factor and prostacyclin [213]. Besides the activation of Ag II, ACE plays a concomitant role in the regulation of hypertension via the inactivation of an endothelium-dependent vasodilatory peptide, bradykinin [214].

The need to regulate hypertension has led to the development of ACE inhibitors. To date, different classes of synthetic ACE inhibitors are available on the market, such as captopril, lisinopril, and enalapril. This class of drugs has demonstrated clinical efficacy in terms of treating hypertension and reducing mortality [215,216]. Although, treatments with ACE inhibitors have also been associated with unpleasant or intolerable side effects, including hypotension, angioedema, coughing, dizziness, headaches, nausea, and renal damage [217]. Synthetic antihypertensive drugs also represent a major burden on global healthcare costs. Thus, there is great interest in the use of antihypertensive compounds from natural resources.

### 7.2. Peptides

Food-derived bioactive peptides have demonstrated their antihypertensive properties through the inhibition of enzymes involved in the regulation of mammalian blood pressure, e.g., ACE and renin [217,218]. In addition, certain peptides have demonstrated the ability to enhance the endothelial nitric oxide synthase pathway, leading to an increase in nitric oxide levels within the vascular walls, thus promoting vasodilation [219]. Other peptides were reported to block the interactions between Ag II, a vasoconstrictor, and angiotensin receptors, which contributes to reducing blood pressure [220]. However, ACE-inhibitory activity is the most-studied metabolic pathway for the development of antihypertensive peptides.

Various sources of plant and animal proteins, mainly from dairy products and fish, have been used to produce ACE-inhibitory peptides (for a review, see Monroe and Fitzgerald [221]), but the use of microalgal proteins has been very limited. Thus far, *Chlorella vulgaris*, *C. ellipsiodea, Arthrospira platensis*, and *Nannochloropsis oculata* have been used to produce ACE-inhibitory peptides [193]. Their effectiveness in lowering blood pressure has been reported extensively using both in vitro and animal studies. A purified peptide isolated from the pepsin hydrolysate of algae protein waste from *Chlorella vulgaris*, with the amino acid sequence Val-Glu-Cys-Tyr-Gly-Pro-Asn-Arg-Pro-Gln-Phe, was found to possess ACE-inhibitory activity with an IC_50_ = 29.6 μM [222]. Two purified peptides, Gly-Met-Asn-Asn-Leu-Thr-Pro and Leu-Glu-Gln, from the cultured marine microalga, *Nannochloropsis oculata,* showed ACE inhibitory activity with IC_50_ values of 123 μM and 173 μM, respectively [119]. Similarly, a potent ACE inhibitory peptide from the marine *Chlorella ellipsoidea* composed of only four amino acids, Val–Glu–Gly–Tyr, was reported to have ACE inhibitory activity (IC_50_ value = 128.4 μM) as well as an antihypertensive effect in spontaneously hypertensive rats (SHR) when administrated orally [223].

The intensity of the antihypertensive effect is strongly influenced by the structure and composition of the bioactive peptides [224]. Results of analytical and chemometric experiments showed that ACE-inhibitory peptides preferred to bind to C-terminal or N-terminal catalytic sites of ACE [225]. Moreover, it was suggested that substrates containing amino acids with cyclic or aromatic rings (e.g., Pro, Phe, Tyr, Trp) at their C-terminus and hydrophobic amino acids, especially those with aliphatic chains (e.g., Gly, Ile, Leu, Val), at their N-terminus were thought to enhance ACE-inhibition [226,227]. Despite evidence citing the importance of the C-terminal domain in the role of blood pressure regulation, the N-terminal domain was deemed the most important for ACE inhibition; the benefits consisted mainly of higher blockage of Angiotensin II production and better prevention of organ damage [228].

## 8. Microalgal Compounds with Anti-Inflammatory Properties

### 8.1. Inflammation in the Body

Inflammation is a natural defense mechanism against pathogens. Although, chronic episodes of inflammation may cause renal, neurodegenerative, and cardiovascular diseases, and it is a leading cause of cancer, diabetes, and obesity [229]. There is increasing evidence that oxidative stress is the common factor underlying several inflammatory pathways, e.g., an increase in reactive oxygen species (ROS) has been suggested to be directly involved in Alzheimer’s disease. This is supported by the overproduction of ROS in early-stage patients that results in cleaving and misfolding of the amyloid precursor protein to amyloid beta-peptide (Aβ), which induces mitochondrial dysfunction and facilitates the formation of amyloid plaques [230]. The disease pathology upregulates pro-inflammatory cytokines and free radical generation, which subsequently stimulates the production of Aβ peptide and other inflammatory pathways [231]. Additionally, ROS generated in brain tissues can modulate synaptic and non-synaptic communication between neurons that may result in neuroinflammation and cellular death, and subsequently in neurodegeneration and memory loss [232].

Results from epidemiological and experimental studies strongly suggest that the use of medicinal plants or dietary supplements with bioactive plant-based extracts can have anti-inflammatory effects [233]. Contradictory results have been also reported [234]. Similarly, natural products from microalgae have been investigated for their anti-inflammatory properties. The most studied compounds are carotenoids, PUFAs, and modified carbohydrates [229,235]. However, there are still many other microalgal metabolites that are also reported to suppress chronic inflammation and alleviate disease-associated symptoms, including phycobiliproteins, phenolic compounds, and various terpenoids [229,236].

Algal extracts have exhibited anti-inflammatory activities, notably through the inhibited production of pro-inflammatory cytokines and eicosanoids and the reduced expression of pro-inflammatory genes [237]. The mechanisms of action from these algal metabolites are very diverse and include the modulation of enzyme activities (e.g., phospholipase A2, cyclooxygenase-2 (COX2), nitric oxide synthase (NOS)), regulation of cellular activities, and the interference of two major signaling pathways [238]. These include the nuclear factor κB (NF-κB) and the mitogen-activated protein kinase pathways, which play a key role in the production of various proinflammatory mediators. Algal compounds in extracts may also have anti-inflammatory properties combined with their antioxidant properties [239].

### 8.2. Omega Fatty Acids

Long-chain PUFAs, including EPA and DHA, are reported to have a therapeutic role in a variety of inflammatory pathologies, such as Alzheimer’s disease, arthritis, and lupus [240]. A growing number of studies in animal and in vitro trials support the anti-inflammatory properties of microalgal oils. Nauroth et al. [86] reported that the use of docosapentaenoic acid (DPA), another omega-3 PUFA, from *Schizochytrium* sp. inhibited the lipopolysaccharide (LPS)-stimulated secretion of interleukin (IL)-1 beta and tumor necrosis factor-alpha (TNF-α) in human peripheral blood mononuclear cells. The authors also found that rats fed an algal oil containing DPA (16% of the total fatty acids) and DHA (40% of the total fatty acids) significantly reduced inflammation in paw edema model rats in comparison to the control [86]. Banskota et al. [241,242] also reported anti-inflammatory effects in RAW 264.7 macrophage cells from lipid extracts containing monogalactosyldiacylglycerols from *Tetraselmis chuii*, *Chlorella sorokiniana*, and *Chondrus crispus*. Furthermore, the use of microalgal oils in the diet, especially those that are rich in omega-3 fatty acids, has been demonstrated to induce chemopreventative effects on azoxymethane-induced colonic aberrant crypt foci functions [243].

Of note, macroalgal lipids have also been explored for their effects, and Khan et al. [244] reported the anti-inflammatory properties of omega-3 fatty acids from the edible brown seaweed, *Undaria pinnatifid;* stearidonic acid (SDA) was active against phorbol myristate acetate-induced ear inflammation while EPA was active against edema, erythema, and blood flow in mice. Vo et al. [245] demonstrated that PUFAs from the brown seaweed, *Ishige okamurae*, were involved in alleviating inflammation due to an allergic response by reducing histamine release and modulating inflammatory cytokine production in human basophilic KU812F cells. Thus, it is suggested that lipid extracts rich in PUFAs (from micro- or macro-algae) could be beneficial as functional food ingredients to control inflammation.

### 8.3. Carotenoids

Carotenoids are well known for their anti-inflammatory properties. While they are structurally related, they can have diverse mechanisms of action. In particular, astaxanthin has received considerable attention due to its anti-inflammatory properties. Results from in vitro and in vivo studies showed that astaxanthin produced by the microalga, *Haematococcus pluvialis*, possessed inhibitory effects on LPS-induced inflammation [246]. In fact, the anti-inflammatory effects of astaxanthin (dosage of 100 mg/kg) were more pronounced than that of a common anti-inflammatory drug, prednisolone (dosage at 10 mg/kg) [246].

The inhibitory activities of xanthophyll carotenoids have been demonstrated for inducible NOS or COX-2 enzymes in cell lines (e.g., BV2 microglial cells and RAW 264.7 macrophage cells) that were stimulated by LPS [247,248]. Astaxanthin has been reported to inhibit the production of nitrous oxide (NO), prostaglandin E2 (PGL2), and to lower the levels of proinflammatory cytokines, TNF-α, IL-1β, and IL-6 [247,249]. In addition, treatment with astaxanthin at 5 mM was found to improve the phagocytic and microbicide function, and to reduce the oxidative damage to lipids and proteins in human neutrophils [250]. Furthermore, cells pre-incubated with astaxanthin restored expression levels of SHP-1, a small heterodimer protein tyrosine phosphatase, and reduced the expression of NF-κB [249].

Results gathered from animal studies were in accordance with those of in vitro trials. Bennedsen et al. [251] reported that astaxanthin from *H. pluvialis* at 200 mg/kg reduced the levels of gastric inflammation in *Helicobacter pylori*-infected mice following a treatment period of 10 days. The decrease in inflammation and the reduction of mucosa-related bacterial loads in the stomachs of infected mice were attributed to the action of astaxanthin to block the release of interferons, which boosts IL-4 release in splenocytes. A clinical study was also conducted on healthy young women, where it was demonstrated that the ingestion of 2 mg of astaxanthin daily for 8 weeks lowered the C-reactive protein in blood levels [252]. In the same study, astaxanthin was also found to reduce ROS production through downregulation of NF-κB and AP-1 transcription factors, as well as reducing the production of inflammatory cytokines [252].

### 8.4. Polysaccharides

Algae synthesize many structurally-diverse carbohydrates with different types of glycosidic bonds. They can be classified as homo- and hetero-polysaccharides, sulfated polysaccharides (SPS), glyco-proteins/peptides, and pectins [253]. However, the relationship between the structures of polysaccharides and their biological functions are not well characterized because of the diversity and complexity of these polymers, namely the absence of monomeric and structural information [254].

Sulfated polysaccharides (SPS) from micro- and macro-algae are the most studied bioactive group of carbohydrates with anti-inflammatory activity [255]. These compounds can vary widely in their composition, i.e., types of monosaccharides, and their degree of sulfation. Several microalgal genera have been explored for their anti-inflammatory properties, including *Porphyridium* (Rhodophyta), *Phaeodactylum* (Heterokonts), and *Chlorella* (Chlorophyta). Guzman et al. [108] reported that crude polysaccharide extracts from *Chlorella stigmatophora* and *Phaeodactylum tricornutum* demonstrated an anti-inflammatory response in a paw edema test. Of note, SPS from *P. tricornutum* showed a direct stimulatory effect on the immune cells as shown with in vitro and in vivo assays. Similarly, Matsui et al. [256] found that SPS from *Porphyridium* inhibited the migration of leukocytes to the sites of inflammation in vitro, and inhibited the development of erythema in vivo.

Besides microalgae, extensive research exists on the bioactive carbohydrates from seaweeds. Albuquerque et al. [257] showed that heterofucan (sulfated L-fucose) from the brown algae, *Dictyota menstrualis*, decreased inflammation by binding to the cell surface of polymorphonuclear cells, which completely inhibits the migration of the leukocytes into the peritoneal cavity of injured tissue in mice without the production of pro-inflammatory cytokines. In another study, Kang et al. [258], found that fucoidan (sulfated alpha-L-glucan) from *Ecklonia cava* possessed inhibitory effects on the inducible NOS and COX-2 enzymes, which limited the production of NO and PGL2 in RAW 264.7 macrophage cells. Furthermore, Li et al. [259] reported that a fucoidan from *Lamanaria japonica* reduced inflammation of damaged myocardium cells in rats by inactivating the secretion of the cytokines, HMGB1 and NF-κB, two immunoproteins that are secreted during inflammatory responses and necrosis.

## 9. Future Prospects

Within the context of global population growth and the availability of terrestrial food products, microalgae may present reliable and sustainable substitutes to commonly used commodities from animal or plant origins. However, to date, only a limited number of strains, mainly the cyanobacterium, *Arthrospira*, and some green microalgae (e.g., *Chlorella, Dunaliella*, and *Haematococcus*), have been utilized for the commercial production of a rather limited number of products. The shift from a niche market to the widespread use of algal products as food commodities will require extensive research and development. This would include the screening of new species or the improvement of existing strains by genetic engineering and modifications towards the development of microalgae with enhanced production of targeted metabolites. In the end, it is essential that microalgal cultivation and processing can be performed economically to ensure low-cost production that can compete with synthetic or biological production from other organisms.

There has been a growing demand for functional foods and nutraceuticals with targeted health benefits. In this context, the use of protein hydrolysates and bioactive peptides has gained interest for the prevention of illness and the improvement of symptoms from several diseases. Available studies on peptides of microalgal origin, although not very abundant, have provided evidence of promising in vitro biological properties. However, more in vivo and clinical trials are needed to determine the bioavailability of peptides after oral administration, especially since loss of bioactivity may occur due to hydrolysis and/or absorption of peptides in the gastrointestinal tract. In order to maintain the bioactivity of peptides, several strategies are being used, such as microencapsulation, nanoencapsulation, or chemical modification. A better understanding of the structure–activity relationship using bioinformatics should enable the development of peptides with an enhanced bioactivity and help to predict non-desirable effects, such as hemolysis.

## 10. Conclusions

Whole microalgal biomass and extracted high-value co-products are currently used in nutraceuticals and as ingredients in functional foods. It is widely recognized that compounds from microalgal extracts have greater biological and economical value than dried biomass. Additionally, purified compounds are preferable given that there are many undefined biochemicals, potential toxins, and heavy metals in whole microalgal biomass that could be unsafe for consumption and cause serious health conditions. Moreover, literature is needed to inform consumers about the content and acceptability of microalgal products.

The supply of high-value algal products is challenged by the overall costs of production, including cultivation systems and maintenance, limited culture productivity, and the biorefining processes. While open ponds are the most economical choice for large-scale cultivation, they are more prone to contamination, thus closed PBRs are preferred if the selected strains synthesize valuable products that can justify the cost of the system. Harvesting and refining the biomass can also be very expensive, and the costs are largely dependent on the selected processes. Traditional methods for the extraction of high-value metabolites often rely on the use of organic solvents, and residual solvents in the products can compromise health. Thus, greener technologies, like supercritical fluid extraction and enzymatic hydrolysis, are presented as emerging techniques for the isolation of high-value algal products.

Despite the challenges of large-scale cultivation, there is a clear demand for microalgal ingredients in foods, supplements, and potential pharmaceuticals. However, much more research is needed in this field to characterize the biochemical content of candidate microalgae to fully understand their benefits and possible concerns. Research on microalgal protein hydrolysates and purified peptides have demonstrated potential as antioxidants and antihypertensive compounds. Furthermore, given that only a few production strains of microalgae have been investigated (out of more than hundreds of thousands of predicted strains), there are immense opportunities for the discovery of novel bioactive metabolites, including the identification of compounds with potential antimicrobial, antifungal, antitumorigenic, and related activities.

## Figures and Tables

**Table 1:** Major microalgal products and producers for human nutrition (adapted from [12,28]).

Microalgae (form)	Major Producer	Origin	Annual Production (tons/year)	World Production (tons/year)
*Arthrospira* (whole, dried microalgae)	Earthrise Nutraceuticals	USA	450	4100–6700
	Cyanotech Corporation	USA	360	
	Hainan DIC Microalgae	China	350	
	Japan Algae Co., Ltd.	Japan	30–100	
	Parry Nutraceuticals	India	>175	
	FEMICO	Taiwan	50–150	
	Nan Pao International Biotechnology Co. Ltd.	Taiwan	70	
	Biorigin	Switzerland	60	
	TAAU Australia Pty Ltd.	Australia	50–60	
*Chlorella* (whole, dried microalgae)	Taipei	Taiwan	400	2000
	Roquette Klötze	Germany	130–150	
	Blue Biotech	Germany	*	
	Earthrise Nutritionals	USA	*	
β-carotene from *Dunaliella* (as oleoresin)	Cognis Nutrition & Health Co.	Australia	*	1200
	Nature Beta Technology Ltd.	Israel	*	
Astaxanthin from *Haematococcus* (as oleoresin)	Cyanotech Corp.	USA	*	300
	Mera Pharmaceuticals Inc.	USA	*	
	Fuji Chemical Industries	Japan	*	
	BioReal AB	Sweden	*	

* data not available.

**Table 2:** Biochemical composition of different species of microalgae.

Species	Class	% Protein (*w/w*)	% Carbs (*w/w*)	% Fat (*w/w*)	References
*Phaeodactylum tricornutum*	Bacillariophyceae	34.8	16.8	16.1	[45,46]
*Nitzschia closterium*	Bacillariophyceae	26	9.8	13	[45]
*Chlamydomonas reinhardtii*	Chlorophyceae	48	17	21	[47]
*Dunaliella tertiolecta*	Chlorophyceae	11	-	-	[48]
*Dunaliella primolecta*	Chlorophyceae	12	-	-	[49]
*Dunaliella salina*	Chlorophyceae	57	32	6	[47]
*Dunaliella* sp.	Chlorophyceae	34.17	14.57	14.36	[50]
*Scenedesmus obliquus*	Chlorophyceae	48–56	10–17	12–14	[47,51]
*Scenedesmus* sp.	Chlorophyceae	31	28	15	[50]
*Chaetoceros calcitrans*	Coscinodiscophyceae	34	6.0	16	[45]
*Chaetoceros calcitrans*	Coscinodiscophyceae	40	37	23	[52]
*Chaetoceros gracilis*	Coscinodiscophyceae	12	4.7	7.2	[45]
*Chaetoceros muelleri*	Coscinodiscophyceae	59	10	31	[52]
*Skeletonema costatum*	Coscinodiscophyceae	25	4.6	10	[45]
*Thalassiosira pseudonana*	Coscinodiscophyceae	34	8.8	19	[45]
*Spirulina maxima*	Cyanophyceae	60–71	13–16	6-7	[47]
*Synechococcus* sp.	Cyanophyceae	63	15	11	[47]
*Nannochloropsis* sp.	Eustigmatophyceae	30	10	22	[50]
*Nannochloropsis granulata*	Eustigmatophyceae	18–34	27–36	24–28	[46]
*Pavlova* sp.	Pavlovophyceae	24–29	6–9	9–14	[45,47]
*Porphyridium cruentum*	Porphyridiophyceae	28–39	40-57	9–14	[47]
*Tetraselmis chuii*	Prasinophyceae	31–46	25	12	[45,46]
*Tetraselmis* sp.	Prasinophyceae	36	24	-	[53]
*Prymnesium* sp.	Prymnesiophyceae	28–45	25–33	22–38	[54]
*Isochrysis galbana*	Prymnesiophyceae	27	34	11	[55]
*Schizochytrium* sp.	Thraustochytriaceae	-	-	50–77	[56]
*Botryococcus braunii*	Trebouxiophyceae	39–40	19–31	25–34	[46]
*Chlorella pyrenoidosa*	Trebouxiophyceae	57	26	2	[56]
*Chlorella vulgaris*	Trebouxiophyceae	51–58	12–17	14–22	[47]

**Table 3:** Market value of selected high-value products isolated from microalgae compared to whole biomass.

Product	Price (USD kg^−1^)	References
Astaxanthin	2500–7000	[77]
β-carotene	300–1500	[14]
Omega-3 fatty acids	80–160	[14]
*Chlorella* biomass	44	[78]
*Arthrospira* biomass	42 *	[79]

* Value expressed in € by the authors; the value shown was converted to USD ($1.17 USD/€).

**Table 4:** Bioactive compounds from microalgae and their potential health benefits.

Bioactive Compounds	Source	Health Benefits	References
**Carotenoids**			
**β-carotene**	*Dunaliella salina*	Antioxidant, pro-vitamin A, anti-allergic, anti-inflammatory	[89,90]
**Astaxanthin**	*Haematococcus pluvialis, C. zofingiensis*	Antioxidant, anti-inflammatory	[91]
**Lutein**	*Scenedesmus spp., Muriellopsis sp., C. sorokiniana*	Antioxidant, anti-inflammatory	[92,93,94]
**PUFAs**			
**Arachidonic acid** **(AA)**	*Porphyridium purpureum*, *P. cruentum,Parietochloris incisa*	Improves normal growth, visual and functional development in infants	[95,96,97]
**Eicosapentaenoic acid (EPA)**	*Nannochloropsis* sp., *Phaeodactylum tricornutum*, *Porphyridium cruentum*	Cardiovascular benefits, mental development and support, anti-inflammatory, protection against atherosclerosis	[98,99,100]
**Docosahexaenoic acid (DHA)**	*Crypthecodinium cohnii*, *Schizochytrium* spp., *Ulkenia* spp.	Cardiovascular benefits, improves nervous system development and function of the brain	[101,102]
**Other metabolites**			
**Peptides**	*Chlorella pyrenoidosa,* *Nannochloropsis oculata*	Antioxidant, anti-inflammatory, anticancer, antihypertensive	[103,104]
**Phenolics**	*Arthrospira maxima, Tetraselmis suecica, Botryococcus braunii,* *Isochrysis* sp., *Chlorella vulgaris, Nannochloropsis* sp.	Antioxidant	[22,105,106]
**Phycocyanin**	*Arthrospira platensis*	Antioxidant, anti-inflammatory	[107]
**Sulfated polysaccharides**	*C. pyrenoidosa,C. stigmatophora*, *Porphyridium* sp., *Phaeodactylum tricornutum*	antioxidant, anti-inflammatory, antiviral, immunomodulatory	[108,109,110]
**“Water-soluble extract”**	*Chlorella stigmatophora, Phaeodactylum tricornutum, Graesiella* sp.	Anti-inflammatory, analgesic, antioxidant, antiproliferative	[108,111]
